# Influence of Hemostatic Disorder on Type II Endoleak Development After Endovascular Abdominal Aortic Aneurysm Repair

**DOI:** 10.3390/ijms27073288

**Published:** 2026-04-04

**Authors:** Paweł Rynio, Magdalena Kłysz, Rabih Samad, Marta Bieniek, Dagmara Lisman, Anita Rybicka, Patryk Skórka, Paulina Lempek, Miłosław Cnotliwy, Arkadiusz Kazimierczak, Piotr Gutowski, Maria Jastrzębska, Aldona Siennicka

**Affiliations:** 1 Department of Vascular Surgery, Pomeranian Medical University, 70-111 Szczecin, Poland; 2 Department of Medical Analytics, Pomeranian Medical University, 70-111 Szczecin, Poland; 3 Department of Genomics and Forensic Genetics, Pomeranian Medical University, 70-111 Szczecin, Poland; 4 Department of Nursing, Faculty of Health Sciences, Pomeranian Medical University, 71-210 Szczecin, Poland

**Keywords:** type II endoleak, hemostasis, thrombin generation, PAI-1, intraluminal thrombus

## Abstract

Endovascular aneurysm repair (EVAR) is a widely used minimally invasive treatment for abdominal aortic aneurysms. However, postoperative type II endoleak (T2EL) remains a relevant complication associated with a risk of aneurysm rupture and the need for repeated imaging follow-up, resulting in exposure to ionizing radiation. Identification of biological factors predisposing to T2EL may improve risk stratification. This pilot study aimed to investigate whether disturbances in hemostasis are associated with early T2EL development after EVAR. A total of 103 patients treated with EVAR for symptomatic or asymptomatic abdominal aortic aneurysms in a tertiary vascular center were prospectively enrolled. Blood samples were collected preoperatively and one month postoperatively to assess fibrinogen, prothrombin fragment F1+2 (F1+2), thrombin–antithrombin complex (TAT), tissue plasminogen activator antigen (tPA), plasminogen activator inhibitor-1 (PAI-1) activity, and platelet activity. Computed tomography angiography (CTA) during follow-up was used to detect endoleaks and calculate their volume. Patients with T2EL had significantly lower levels of prothrombin fragment F1+2 and higher PAI-1 activity compared with patients without endoleak. No significant association was observed between the analyzed biomarkers and endoleak volume. These findings suggest that reduced thrombin generation and impaired fibrinolysis may contribute to endoleak formation after EVAR and warrant further investigation in larger, confirmatory studies.

## 1. Introduction

Abdominal aortic aneurysms (AAAs) are a prevalent condition among the elderly population, particularly affecting men aged 55 and older, with an estimated prevalence ranging from 4% to 8.9% [1,2,3]. This condition is associated with a substantial mortality risk and represents the tenth leading cause of death in this population [4]. Endovascular aneurysm repair (EVAR) is a widely used treatment for AAA and involves implanting a stent-graft into the aorta and aneurysm sac to prevent rupture and related fatal outcomes [5].

Despite the effectiveness of EVAR, endoleaks (ELs), defined as persistent blood flow into the aneurysm sac after stent-graft implantation, remain a frequent complication, occurring in up to one-third of patients [6]. The presence of an EL is associated with an increased risk of aneurysm pressurization and rupture. The reported rupture risk for type II endoleaks (T2EL) is relatively low, approximately 1% [7]. T2EL is the most common EL subtype, occurring in approximately one in five patients at 1 month after EVAR. Importantly, more than half of T2ELs regress spontaneously within 6 months of follow-up [8]. Current management of T2EL relies primarily on surveillance with serial computed tomography angiography (CTA), with conservative treatment as the initial strategy. Additional endovascular interventions are considered when the EL persists or is accompanied by aneurysm sac enlargement [9]. However, this approach requires repeated imaging, exposing patients to cumulative ionizing radiation and contrast agents, underscoring the need for improved risk stratification strategies.

To the best of our knowledge, most previous studies have focused primarily on anatomical predictors of T2EL, including the number of lumbar arteries (LAs) supplying the aneurysm sac and patency of the inferior mesenteric artery (IMA) [10]. In contrast, the potential contribution of biological factors, particularly those related to thrombin generation, fibrinolysis, and platelet activity, remains unclear. Early identification of patients at risk for EL after EVAR is crucial for targeted surveillance and management. The identification of circulating, biologically active markers associated with EL development may facilitate such stratification while also reducing healthcare costs, radiation exposure, and contrast-related complications.

Therefore, this study aimed to identify hemostatic disorders associated with EL occurrence. We assessed the relationship between T2EL development and selected hemostatic markers, including fibrinogen, prothrombin fragment F1+2 (F1+2), thrombin-antithrombin complex (TAT), tissue plasminogen activator antigen (tPA), plasminogen activator inhibitor-1 (PAI-1) activity, and platelet activity. These potential biomarkers have previously demonstrated diagnostic potential for predicting venous thromboembolism, providing a rationale for their evaluation in the context of post-EVAR EL occurrence [11].

## 2. Results

The study included 103 patients, of whom 98 (95%) were male. Clinical characteristics are presented in Table 1. During the study period, early T2EL was detected on one-month follow-up CTA in 17 patients (17%). There was no significant difference in aneurysm diameter between the endoleak-free and endoleak groups (61.1 mm vs. 61.7 mm). However, the number of LAs originating from the aneurysm sac was significantly higher in patients with T2EL compared with those without T2EL (mean 3.58 vs. 5.41; *p* = 0.001).

Additional anatomical and treatment-related variables did not differ significantly between patients with and without T2EL. Patent IMA was observed in 11 of 17 patients with T2EL (64.7%) and in 53 of 86 patients without T2EL (61.6%) (*p* = 1.000). Anticoagulation therapy was used in 3 patients with T2EL (17.6%) and in 18 patients without T2EL (20.9%) (*p* = 1.000).

Table 2 presents an analysis of selected plasma–platelet hemostatic parameters measured before surgery and one month after EVAR. Preoperative F1+2 measurements were available for 94 of 103 patients (77 endoleak-free and 17 with T2EL). Subsequent biomarker analyses and logistic regression analyses were performed in this subset. F1+2 levels and PAI-1 activity differed significantly between the endoleak-free and endoleak groups. Specifically, F1+2 levels were lower, whereas PAI-1 activity was higher in patients with T2EL. No significant differences were observed between preoperative and postoperative measurements within groups. Additional plasma and platelet hemostatic parameters are presented in the Supplementary Materials (Table S1).

**Figure 1:**
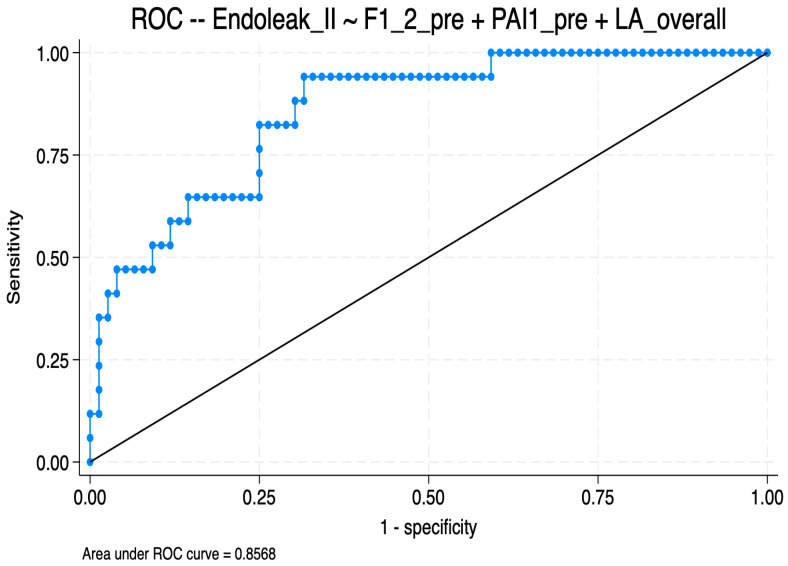
Receiver operating characteristic (ROC) curve for the multivariable logistic regression model predicting type II endoleak (n = 93). Area under the curve (AUC) = 0.857.

**Figure 2:**
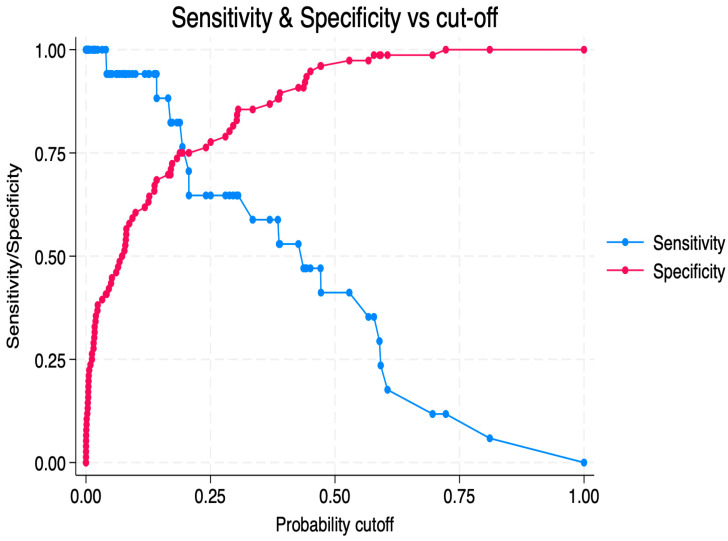
Sensitivity and specificity across predicted probability cut-off values for the multivariable logistic regression model predicting type II endoleak (n = 93). The optimal threshold determined by the Youden index was 0.14, corresponding to a sensitivity of 94.1% and a specificity of 68.4%.

An exploratory multivariable logistic regression model was constructed, including the number of LAs, baseline F1+2 levels, and baseline PAI-1 activity. Given the limited number of T2EL events, the number of predictors included in the model was intentionally restricted. The results of the multivariable logistic regression analysis are shown in Table 3. In this exploratory model, the ROC curve showed moderate discriminatory performance, with an area under the curve (AUC) of 0.857 (Figure 1). At the optimal probability threshold determined by the Youden index (0.14), the model achieved a sensitivity of 94.1%, a specificity of 68.4%, a positive predictive value of 40.0%, and a negative predictive value of 98.1% (Figure 2). At the default threshold of 0.50, specificity was 97.4% and sensitivity was 41.2%, with an overall accuracy of 87.1%. No significant correlations were observed between F1+2 levels, PAI-1 activity, and T2EL volume or surface area.

## 3. Discussion

Endoleaks remain one of the most frequent and clinically relevant complications following EVAR, with T2EL being the most common subtype. Although T2EL is often regarded as a benign phenomenon, its persistence may prevent aneurysm sac regression and, in selected cases, contribute to sac enlargement and an increased risk of rupture [12]. Among established predictors of T2EL, anatomical factors play a dominant role, particularly IMA patency and the number of patent LAs [10]. Previous studies have reported that, in addition to IMA patency, advanced chronic kidney disease and the presence of ≥4 patent LAs are independently associated with a significantly increased risk of T2EL [13].

Despite the well-established role of anatomical determinants, no optimal circulating biomarker has been identified for predicting EL development or persistence. This suggests that mechanisms beyond vascular anatomy may influence post-EVAR aneurysm sac behavior. Increasing evidence indicates that biological processes within the aneurysm sac and wall play a critical role in post-EVAR remodeling. Experimental studies have also reported that vascular graft implantation may stimulate the local production of growth factors such as platelet-derived growth factor (PDGF) and basic fibroblast growth factor (bFGF), which promote vascular smooth muscle cell (VSMC) proliferation and vascular wall remodeling [14].

Moreover, proteomic analysis of the aortic wall after EVAR has shown downregulation of structural and metabolic proteins, including collagen types I and III, actin, and tropomyosin, as well as proteins involved in energy production. These alterations may impair VSMC contractility, extracellular matrix (ECM) integrity, and tissue repair capacity, thereby potentially affecting thrombus formation and stabilization within the aneurysm sac [15].

In this context, the present study aimed to determine whether disturbances in systemic hemostasis are associated with T2EL. Previous clinical observations suggest that pharmacological interference with thrombus formation and stabilization may promote EL persistence. Specifically, Kong et al. observed that combined antithrombotic therapy after EVAR was associated with a higher risk of T2EL compared with antiplatelet therapy alone, indicating that impaired thrombus formation may favor sustained collateral perfusion. Similarly, dual antiplatelet therapy has been reported to inhibit intraluminal thrombus (ILT) formation and maintain patency of collateral vessels such as LAs and the IMA. These observations provided the rationale for investigating selected hemostatic markers as potential predictors of EL development in the present study [16]. In our cohort, IMA patency and anticoagulant therapy did not differ significantly between patients with and without T2EL, although both variables remain clinically important confounders.

In this study, lower preoperative levels of prothrombin fragment F1+2 and higher PAI-1 were independently associated with T2EL in the multivariable model. Additionally, a higher number of LAs supplying the aneurysm sac remained an independent anatomical predictor. Together, these findings suggest that reduced thrombin generation, impaired fibrinolytic remodeling, and increased collateral inflow may synergistically contribute to T2EL persistence.

**Figure 3:**
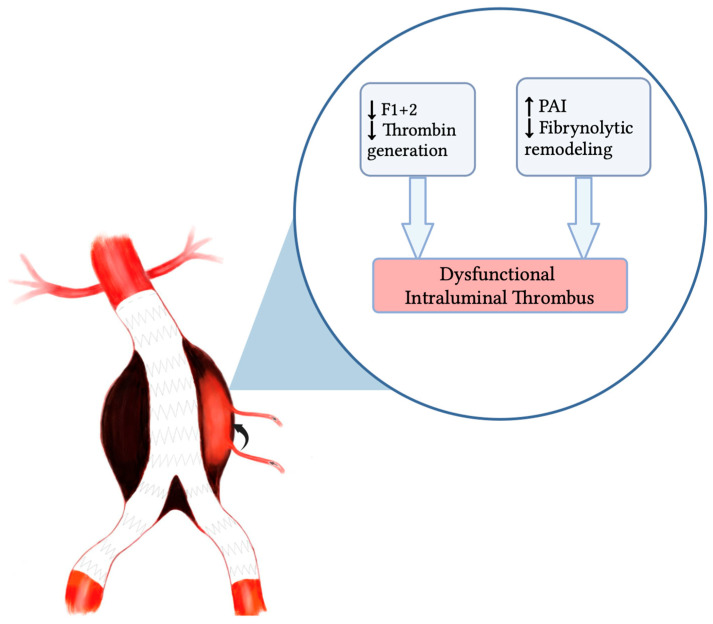
Proposed mechanistic model of hemostatic dysregulation contributing to T2EL persistence post-EVAR. Reduced thrombin generation (↓F1+2) and impaired fibrinolytic remodeling (↑PAI-1) may lead to dysfunctional ILT, limiting effective sealing of collateral vessels.

At first glance, the coexistence of reduced thrombin generation and suppressed fibrinolysis may appear contradictory. However, these findings reflect distinct and complementary components of hemostasis and together define a coherent pathological phenotype. Prothrombin fragment F1+2 is a direct marker of thrombin generation and reflects activation of the prothrombinase complex (factor Xa/Va). Sufficient thrombin generation is required for the formation of a compact and mechanically stable ILT capable of sealing the aneurysm sac and limiting retrograde collateral flow. Moreover, increasing evidence indicates that ILT is not a passive structure, but a biologically active matrix involved in inflammatory signaling, activation of matrix metalloproteinases (MMP-2 and MMP-9), and ECM turnover [17]. Enhanced factor Xa activity has been shown to promote MMP activation and pro-inflammatory cytokine release, contributing to controlled remodeling of the aneurysm wall [18] (Figure 3).

However, these mechanisms should be interpreted with caution, as the present study assessed systemic hemostatic markers rather than biological processes within the aneurysm sac.

Accordingly, reduced F1+2 levels observed in patients with T2EL indicate reduced factor Xa-dependent thrombin generation, which may result in the formation of an incompletely organized ILT. Such a thrombus may be less effective in sealing collateral inflow from LAs and the IMA, thereby permitting persistent, low-pressure retrograde blood flow within the aneurysm sac. Persistent collateral perfusion may therefore sustain biological activity and potentially influence local remodeling processes.

These findings are partially consistent with previous research linking hemostatic factors to EL behavior. Sugimoto et al. reported that elevated D-dimer levels were associated with aneurysm sac enlargement during long-term follow-up in patients with persistent T2EL, suggesting ongoing thrombus turnover [19]. Moreover, Bailey et al. demonstrated a systemic hypercoagulable state following EVAR, characterized by elevated levels of TAT, F1+2, and D-dimer, in contrast to open repair [20]. However, in this cohort, these parameters did not differ significantly between patients with and without EL [21]. This observation supports the concept that local, rather than systemic, dysregulation of coagulation and fibrinolysis may be more relevant to the development of T2EL.

Interestingly, aneurysm diameter did not differ between patients with and without EL, suggesting that factors other than sac size may play a more crucial role in T2EL persistence. Instead, local hemodynamics, graft integrity, and collateral vessel anatomy may play a more critical role [8]. From a clinical perspective, integrating potential hemostatic biomarkers with anatomical assessment may improve risk stratification before EVAR. Measurement of prothrombin fragment F1+2 and PAI-1 activity, together with assessment of IMA patency and LA anatomy, could help identify patients at higher risk of developing T2EL. If confirmed in larger prospective studies, such an approach may support more individualized surveillance strategies and help identify anatomically suitable high-risk patients for consideration of adjunctive preventive procedures, such as selective embolization of the IMA or patent LAs. Therefore, understanding the role of hemostatic factors in EL development may open new avenues for more individualized preventive and therapeutic strategies.

## 4. Materials and Methods

### 4.1. Patients

This prospective study included consecutive patients with symptomatic or asymptomatic AAAs who underwent EVAR at the Department of Vascular, General, and Angiological Surgery, Pomeranian Medical University in Szczecin. Before surgery, venous blood was collected from the superficial veins of the ulnar fossa to measure fibrinogen, F1+2, TAT, tPA, PAI-1, and platelet activity. Laboratory analyses were performed at the Department of Medical Analytics and the Department of Laboratory Diagnostics, Pomeranian Medical University in Szczecin. Follow-up CTA was performed one month after EVAR, in accordance with the recommendations of the European Society for Vascular Surgery (ESVS) [22]. During the follow-up visit, venous blood samples were collected again for repeat assessment of the same hemostatic parameters. The cohort was enrolled between 2018 and 2020 and consisted of patients with and without T2EL after EVAR.

### 4.2. Imaging Analysis

CTA scans were evaluated by a vascular surgeon with 12 years of experience in CTA image analysis. Anatomical data obtained from CTA imaging were documented, including aneurysm diameter, the number of LAs originating from the aneurysm sac, and the presence of a patent IMA. On follow-up scans, the presence or absence of EL was recorded. When an EL was identified, CTA scans were imported into 3D Slicer, and semi-automated tools were used to segment the EL [23]. Segmentation and volumetric analysis were performed by a single observer. The segmented regions enabled quantitative assessment of T2EL volume and surface area. These data were subsequently correlated with serum hemostatic parameters. For multivariable logistic regression analysis, prothrombin fragment F1+2 values were scaled per 100 units to improve the interpretability of the estimated odds ratios (ORs).

### 4.3. Statistical Analysis

Clinical and demographic data of the study groups were summarized as counts and frequencies, with continuous variables expressed as means ± standard deviations (SD). Similarly, hemostatic parameter levels and EL volumes were reported as means ± SD. Differences between groups were compared using Student’s *t*-test for normally distributed variables or the Mann–Whitney U test for non-normally distributed variables. Data normality was assessed using histograms and the Shapiro–Wilk test. Correlations between EL volume and hemostatic parameter levels were assessed using Pearson or Spearman correlation coefficients, depending on the data distribution. Correlation analysis was performed only for variables that showed statistically significant differences between the endoleak and no-endoleak groups. Categorical variables were compared using Fisher’s exact test due to the small number of observations in the EL group. Variables showing statistically significant differences between groups were included in a multivariate logistic model. To minimize the risk of model overfitting given the limited number of T2EL events, the number of predictors was intentionally restricted, and variables were selected based on prior biological relevance and statistical significance in univariable comparisons. The multivariable model should therefore be considered exploratory and hypothesis-generating. Model performance was assessed using a receiver operating characteristic (ROC) curve. The optimal probability cut-off was determined using the Youden index, and the corresponding sensitivity, specificity, positive predictive value, and negative predictive value were calculated. A *p*-value of <0.05 was considered statistically significant.

## 5. Limitations

However, our study has several limitations that need to be acknowledged. First, our sample size was relatively small and derived from a single center, which limits the generalizability and statistical power of our findings. In addition, the relatively small number of T2EL events (n = 17) may limit the robustness of multivariable analyses and increase the risk of model instability. Therefore, the results of the multivariable logistic regression model should be interpreted with caution and considered exploratory. Second, this study design was prone to selection bias, as we enrolled consecutive patients from a specific hospital department. Third, our follow-up duration was limited to one month, which may not capture the long-term dynamics of endoleak occurrence and resolution. Early T2ELs detected at this time point may resolve spontaneously during subsequent follow-up, while others may develop later. Therefore, a single early CTA assessment may not fully reflect the long-term behavior of T2ELs. Fourth, we did not perform serial biomarker measurements at multiple time points, which could provide more information on the temporal changes in hemostatic factors. Fifth, we used a semi-automated segmentation method for endoleak detection and quantification, which required manual adjustments and may introduce variability. Sixth, we focused on specific hemostatic biomarkers and did not explore other factors that may contribute to endoleak pathophysiology, such as inflammation, oxidative stress, and endothelial dysfunction. Seventh, selected additional potential confounding factors, including IMA patency and anticoagulation therapy, were assessed descriptively. These variables were not included in the exploratory multivariable model because of the limited number of T2EL events. Therefore, residual confounding cannot be excluded.

## 6. Conclusions

These findings highlight the potential value of integrating hemostatic biomarkers with anatomical assessment to improve risk stratification in patients undergoing EVAR. If validated in larger prospective studies, such biomarkers may support more individualized surveillance strategies and could help identify high-risk patients who may benefit from further evaluation of adjunctive preventive approaches, including selective embolization of the IMA or patent LAs in anatomically suitable cases.

## Figures and Tables

**Table 1:** Baseline clinical characteristics of the study population.

	Endoleak (+) n = 17	Endoleak (−) n = 86	*p* Value
Male sex	16 (94%)	82 (95%)	1.000
Diabetes mellitus	3 (18%)	25 (29%)	0.390
Coronary artery disease	3 (18%)	42 (49%)	0.030
Myocardial infarction	3 (18%)	28 (33%)	0.262
Stroke	3 (18%)	5 (7%)	0.124
Hypertension	13 (76%)	70 (81%)	0.738
Current or former smoking	2 (12%)	6 (7%)	0.616

Values are presented as n (%).

**Table 2:** Selected hemostatic parameters associated with type II endoleak (T2EL).

Hemostatic Parameter	Endoleak (+), n = 17 Mean ± SD	Endoleak (−), n = 77 Mean ± SD	*p* Value
Prothrombin fragment F1+2 (baseline)	3467 ± 1947	7907 ± 7960	0.016
Prothrombin fragment F1+2 (1 month)	4013 ± 2734	9153 ± 9364	0.027
PAI-1 activity (baseline)	23.5 ± 15.7	16.5 ± 17.9	0.020
PAI-1 activity (1 month)	23.0 ± 14.4	14.8 ± 14.2	0.016

Values are presented as mean ± SD.

**Table 3:** Multivariable logistic regression analysis for type II endoleak (T2EL) occurrence.

Parameter	OR (95% CI)	*p* Value
Prothrombin fragment F1+2 (per 100 units, baseline) *	0.98 (0.95–1.00)	0.041
PAI-1 activity (baseline)	1.05 (1.01–1.09)	0.008
Number of lumbar arteries	2.06 (1.29–3.27)	0.002

Multivariable model: n = 93, pseudo R^2^ = 0.291; Hosmer–Lemeshow χ^2^(8) = 5.90, *p* = 0.659. OR, odds ratio. * OR per 100 pmol/L increase. OR, odds ratio; CI, confidence interval.

## Data Availability

The data presented in this study are available from the corresponding author upon reasonable request. The data are not publicly available due to ethical and privacy restrictions.

## Supplementary Materials

The following supporting information can be downloaded at: https://www.mdpi.com/article/10.3390/ijms27073288/s1, Table S1: Plasma and platelet hemostatic parameters in patients with and without type II endoleak (T2EL).

## Abbreviations

The following abbreviations are used in this manuscript:

AAA	Abdominal Aortic Aneurysm
ADP	Adenosine Diphosphate
ASPI	Arachidonic Acid-induced Platelet Aggregation
AUC	Area Under the Curve
bFGF	Basic Fibroblast Growth Factor
CI	Confidence Interval
CRP	C-reactive protein
CTA	Computed Tomography Angiography
ECM	Extracellular Matrix
EL	Endoleak
ELs	Endoleaks
ESVS	European Society for Vascular Surgery
EVAR	Endovascular Aneurysm Repair
F1+2	Prothrombin Fragment F1+2
IMA	Inferior Mesenteric Artery
ILT	Intraluminal Thrombus
LA	Lumbar Artery
LAs	Lumbar Arteries
MMP	Matrix Metalloproteinase
OR	Odds Ratio
PAI-1	Plasminogen Activator Inhibitor-1
PDGF	Platelet-Derived Growth Factor
ROC	Receiver Operating Characteristic
SD	Standard Deviation
T2EL	Type II Endoleak
TAT	Thrombin–Antithrombin Complex
tPA	Tissue Plasminogen Activator Antigen
VSMC	Vascular Smooth Muscle Cell
