# Supplementary material for: Influence of Hemostatic Disorder on Type II Endoleak Development After Endovascular Abdominal Aortic Aneurysm Repair
Source: Int J Mol Sci. 2026 Apr 4;27(7):3288. doi: 10.3390/ijms27073288 (PMC13073863; doi:10.3390/ijms27073288)
Supplement: Supplementary file 1 [file ijms-27-03288-s001.zip › ijms-4130985-supplementary.pdf]

Table S1. Plasma and platelet hemostatic parameters in patients with and without type II endoleak (T2EL).

Parameter	Endoleak (+), n=17 Mean ± SD	Endoleak (-), n=86 Mean ± SD	P value
CRP (mg/L)	8.37 ± 21.60	6.88 ± 12.85	0.301
Fibrinogen (baseline, g/L)	3.86 ± 1.14	4.08 ± 1.49	0.654
Fibrinogen (1 month, g/L)	4.61 ± 1.24	4.80 ± 1.39	0.605
Prothrombin fragment F1+2 (baseline)	3467 ± 1947	7907 ± 7959	0.016
Prothrombin fragment F1+2 (1 month)	4013 ± 2734	9153 ± 9364	0.028
Δ F1+2	546 ± 3538	1262 ± 9182	0.933
Thrombin–antithrombin complex (baseline, ng/mL)	8.11 ± 3.27	7.74 ± 4.11	0.358
Thrombin–antithrombin complex (1 month, ng/mL)	7.08 ± 3.35	7.32 ± 4.29	0.945
Δ TAT	−1.03 ± 2.44	−0.42 ± 2.13	0.250
tPA antigen (baseline, ng/mL)	15.87 ± 10.29	14.74 ± 8.11	0.934
tPA antigen (1 month, ng/mL)	14.77 ± 7.75	14.68 ± 7.99	0.557
Δ tPA	−1.10 ± 5.36	−0.06 ± 6.00	0.992
PAI-1 activity (baseline)	23.45 ± 15.70	16.41 ± 17.81	0.019
PAI-1 activity (1 month)	22.99 ± 14.43	14.76 ± 14.06	0.015
Δ PAI-1	−0.47 ± 6.85	−1.65 ± 14.01	0.534
ADP-induced aggregation (baseline)	458.1 ± 194.1	474.6 ± 249.7	0.774
ADP-induced aggregation (1 month)	511.9 ± 226.6	571.2 ± 272.1	0.428
ASPI-induced aggregation (baseline)	311.4 ± 287.6	328.0 ± 232.8	0.344
ASPI-induced aggregation (1 month)	267.0 ± 217.4	334.2 ± 236.9	0.213
